# Supplementary material for: Structural basis for activation of a diguanylate cyclase required for bacterial predation in Bdellovibrio
Source: Nat Commun. 2019 Sep 9;10:4086. doi: 10.1038/s41467-019-12051-6 (PMC6733907; doi:10.1038/s41467-019-12051-6)
Supplement: Supplementary file 1 — Supplementary Information [file 41467_2019_12051_MOESM1_ESM.pdf]

Supplementary Information

Structural basis for activation of a diguanylate cyclase required for bacterial predation in *Bdellovibrio*

R. W. Meek et al.

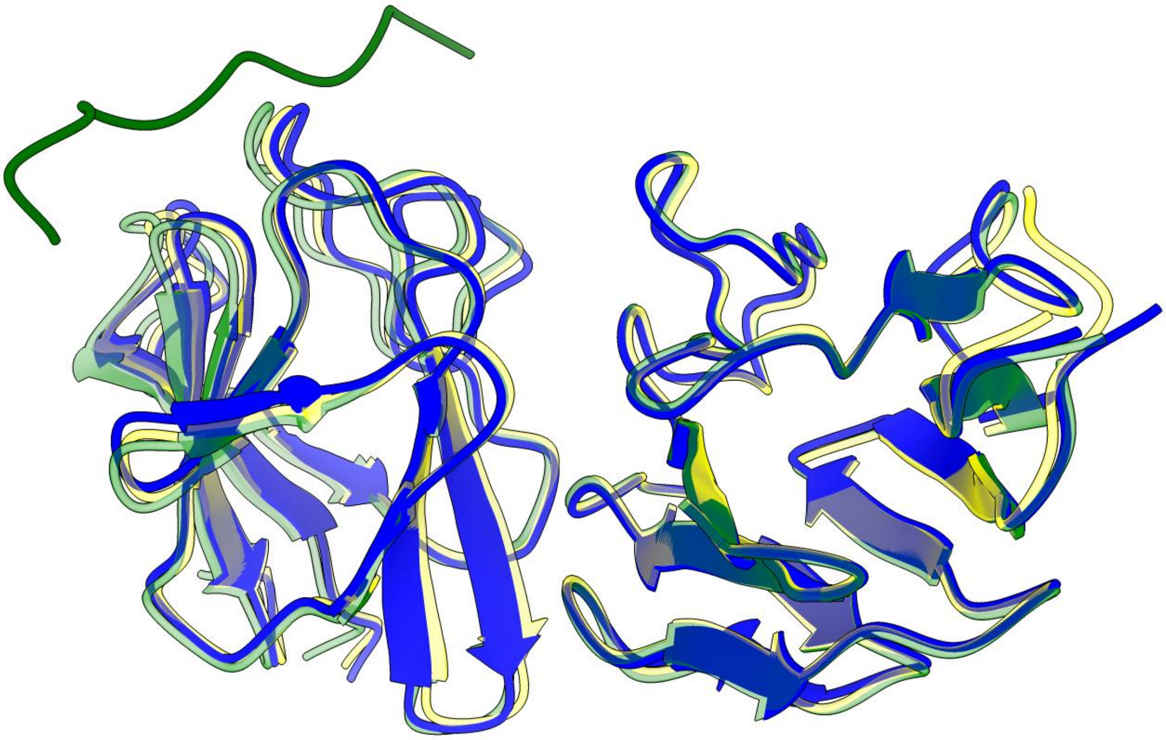

Supplementary Figure 1. Structural Comparison of DgcB Dimers. Upon superposition, it can be observed that all three different DgcB crystal forms utilize a similar FHA:FHA interface (full-length structure, just FHA shown, blue; apo FHA-only form, yellow transparent; phosphopeptide complex, FHA green transparent and phosphopeptide dark green). Despite minor loop perturbations where the phosphopeptide is bound, the dimeric interface remains unaffected.

Construct	Primer Sequence
Full-length (aa 1-310) fwd	5'-gtttaactttaagaaggagatatacatatggctcacaacgatgacaactcag-3'
Full-length (aa 1-310) rev	5'-gctgcactaccgcgtggcacaagcttagcgacgatggtcgtacggttacg-3'
GGDEF domain only (aa 148-310) fwd	5'-ttttgtttaactttaagaaggagatatacatatgcagaaggatgcgttgacgggtgc-3'
GGDEF domain only (aa 148-310) rev	5'-gctgcactaccgcgtggcacaagcttagcgacgatggtcgtacggttacg-3'
FHA domain with N-terminal tail (aa 1-135) fwd	5'-gtttaactttaagaaggagatatacatatggctcacaacgatgacaactcag-3'
FHA domain with N-terminal tail (aa 1-135) rev	5'-gccgctgcactaccgcgtggcacaagaatgctgcctttttcaaggaacttgaag-3'
FHA domain only (aa 33-135) fwd	5'-gtttaactttaagaaggagatatacatatgccgcccgcaattgttgttttgatcg-3'
FHA domain only (aa 33-135) rev	5'-gccgctgcactaccgcgtggcacaagaatgctgcctttttcaaggaacttgaag-3'
T14D mutagenesis fwd	5'-ctcagacaacttagaaaaagacagtattgttgccagcgacac-3'
T14D mutagenesis rev	5'-gtgtcgctggcaacaatactgtctttttctaagttgtctgag-3'
R218A mutagenesis fwd	5'-caccaagctgatcgcctccaacgacttc-3'
R218A mutagenesis rev	5'-gaagtcgttggaggcgatcagcttggtg-3'
E229A, E230A mutagenesis fwd	5'-ctttgcccgttacggtggtgcagcgtttgtgctgctgctttcc-3'
E229A, E230A mutagenesis rev	5'-ggaaagcagcagcacaaacgctgcaccaccgtaacgggcaaag-3'
S73C q5-method mutagenesis fwd	5'-tgatgataaatgtttgagccgctc-3'
S73C q5-method mutagenesis rev	5'-atgtagacttggctttcc-3'
S15C q5-method mutagenesis fwd	5'-agaaaaaacctgtattgttgccag-3'
S15C q5-method mutagenesis rev	5'-aagttgtctgagttgtcatc-3'

Supplementary Table 1. Primer sequences for cloning and mutagenesis.
